# The Complex Role of the Complement C3a Receptor (C3aR) in Cerebral Injury and Recovery Following Ischemic Stroke

**DOI:** 10.3390/cells14181440

**Published:** 2025-09-15

**Authors:** Naseem Akhter, Ateeq Lambay, Reema Almotairi, Abdullah Hamadi, Kanchan Bhatia, Saif Ahmad, Andrew F. Ducruet

**Affiliations:** 1 Department of Translational Neuroscience, Barrow Neurological Institute, Phoenix, AZ 85013, USA; naseem.akhter@barrowneuro.org (N.A.);; 2 Department of Medical Laboratory Technology, Faculty of Applied Medical Sciences, Prince Fahad bin Sultan Chair for Biomedical Research, University of Tabuk, Tabuk 71491, Saudi Arabia; ralmotairi@ut.edu.sa (R.A.); a.aldhafri@ut.edu.sa (A.H.); 3 School of Mathematical and Natural Sciences, Arizona State University, Glendale, AZ 85306, USA; kanchan.bhatia@asu.edu; 4 Department of Neurosurgery, Barrow Neurological Institute, Phoenix, AZ 85013, USA

**Keywords:** complement C3a receptor (C3aR), ischemic stroke, neuroinflammation, temporal phases of brain injury, therapeutic modulation

## Abstract

The Complement C3a Receptor (C3aR) plays a multifaceted role along the varying temporal phases of brain injury following cerebral ischemia. C3aR is a G-protein-coupled receptor (GPCR) that binds to its ligand, C3a an anaphylatoxin generated during activation of the complement cascade. During ischemia, complement is activated as part of the initial inflammatory response, with C3aRs playing a time-dependent role in both brain injury and repair mechanisms. In the acute phase (minutes to hours post-ischemia), C3aR activation promotes the recruitment of immune cells and the release of chemokines and cytokines, driving blood–brain barrier (BBB) permeability and brain edema. During the subacute phase (hours to days post-ischemia), C3aR continues to modulate immune cell activity, worsening secondary brain injury, although emerging evidence suggests that C3aR activation in this phase may also aid in the clearance of cellular debris and cell survival. In the chronic phase (days to weeks post-ischemia), chronically elevated C3aR activity can prolong neuroinflammation and impair recovery, whereas controlled C3aR signaling in the subacute/chronic phase can activate reparative pathways (e.g., microglial phagocytosis, astrocyte trophic support). As a result, targeting the C3aR requires careful timing to optimize its benefits. Given the dual impact of C3aR activation, which serves to exacerbate injury in the acute phase but supports repair beginning in the subacute and chronic phases, a targeted therapeutic approach should focus on context- and time-dependent modulation of the C3a/C3aR axis. This strategy would involve blocking the C3aR during the acute phase to reduce inflammation and BBB breakdown while controlling C3a signaling in later phases to promote tissue repair.

## 1. Introduction

Ischemic stroke affects approximately 7.6 million individuals annually, with a global prevalence exceeding 77 million [1]. Despite significant developments in stroke treatment, over 50% of survivors experience lasting impairment, including motor deficits, aphasia, depression, anxiety, cognitive impairment, epilepsy, often necessitating long-term institutional care [2]. Elucidating the molecular pathways contributing to both ischemic brain injury and functional recovery may pave the way for new therapeutic strategies to durable neurological improvement and reduced public health burden [3,4].

The molecular pathways underlying cerebral ischemia/reperfusion injury remain incompletely understood, but complement activation plays a central role [5,6]. Existing data suggest that complement inhibition enhances functional and neuropathological outcomes in ischemic stroke across various animal models [7,8]. Furthermore, clinical evidence demonstrates accumulation of complement proteins within ischemic brain regions [9,10]. Several strategies have sought to target complement to mitigate ischemic damage, via inhibition of complement component 1 (C1) [11], complement component 3 (C3) [12], complement component 5 (C5) [13], and the membrane attack complex (MAC, C5b-9) [14]. Additionally, cobra venom factor (CVF) [15] and intravenous immunoglobulin (IVIg) have been used to suppress complement activity or eliminate circulating components in preclinical models. Despite these promising experimental results, few anti-complement therapeutics have been successfully developed [16,17,18]. Additionally, emerging evidence suggests that complement contributes significantly to reparative and regenerative processes [19,20,21]. As a result, the complex role of complement activation in cerebral ischemia remains poorly elucidated [22].

A major challenge in stroke therapeutic design lies in the complexity and multifactorial nature of stroke pathology disrupting various homeostatic processes. While animal model studies have enhanced our understanding of stroke pathophysiology and enabled therapeutic screening, successful translation of these findings into the clinic remains elusive. One critical factor is the timing of interventions and inadequate consideration of chronic outcomes.

The complement system is rapidly activated following cerebral ischemia, contributing to secondary injury via neuroinflammation, blood–brain barrier (BBB) disruption, and neuronal death. While multiple complement components are involved in stroke pathology, the C3a/C3a receptor (C3aR) axis represents a central and highly actionable node in this cascade [23]. C3 is the converging point of all three complement pathways (classical, lectin, and alternative), and its cleavage product, C3a, is rapidly generated post-stroke, exerting potent pro-inflammatory and chemoattractant effects via C3aR, which is expressed on microglia, astrocytes, neurons, and endothelial cells [20,24].

Interestingly, C3a/C3aR signaling plays a dual role, it can mediate deleterious responses in acute injury phases by contributing to neuroinflammation, gliosis, and neuronal loss [25] and on the other hand can mediate protective responses in the subacute-to-chronic stages [20]. Notably, C3aR antagonism in preclinical models of stroke demonstrated reduced infarct volume, preserved BBB integrity, attenuated glial activation, and improved neurological outcomes, underscoring its translational potential [20,23]. Unlike terminal complement components (e.g., C5a or MAC), which act downstream and are harder to control without systemic immunosuppression [26], targeting C3aR offers a more specific modulation of inflammation with reduced risk of broad immune compromise.

Thus, while all complement components participate in ischemic injury, the C3a/C3aR axis stands out due to its upstream position, widespread receptor expression in the CNS, and evidence of its pivotal role in orchestrating post-stroke inflammatory cascades. Selective targeting of the C3aR could therefore achieve neuroprotection without fully abrogating the complement’s protective roles in debris clearance and neurodegeneration.

**Figure 1:**
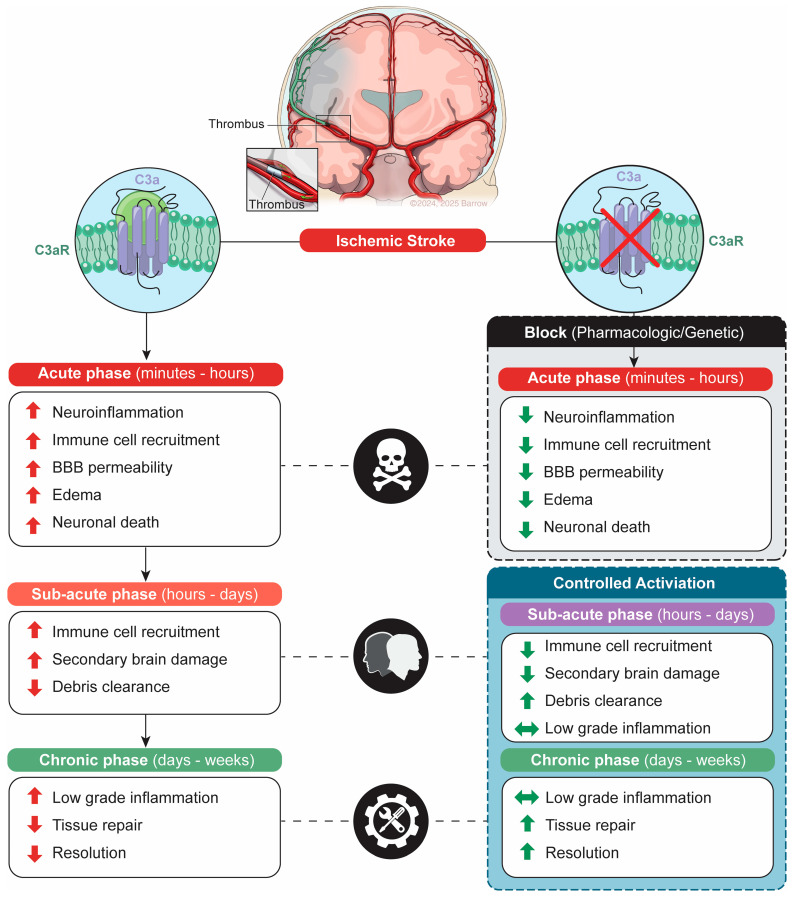
Schematic picture demonstrating the inhibition of C3a receptor (C3aR) signaling in the acute phase and controlled activation in the subacute and chronic phase is neuroprotective in ischemic stroke.

This review synthesizes emerging evidence on the role of the C3aR in the pathophysiology of ischemic stroke, with particular emphasis on its temporally distinct effects across acute and subacute phases. We critically examine C3aR antagonism, a preclinical strategy that modulates complement signaling as a potential therapeutic avenue (Figure 1). By integrating mechanistic insights with translational perspectives, we aim to inform the rational design of complement-based interventions for ischemic stroke recovery.

## 2. The Double-Edged Sword: Microglial and Astrocytic C3/C3a Signaling in Stroke and Neurodegeneration

Traditionally viewed as an immune-privileged site, the central nervous system (CNS) is functionally compartmentalized by the blood–brain barrier (BBB), and remains largely insulated from peripheral complement activity, as the BBB restricts the entry of cellular and humoral elements, including soluble complement proteins. Despite peripheral restrictions, various CNS-resident cells are capable of producing the entire complement cascade locally, with increased expression observed both with normal aging and in neurodegenerative disease in humans and mouse models [27,28,29].

Complement component C3a and its receptor, C3aR, have emerged as critical modulators of neuroinflammation and glial reactivity following ischemic stroke, with distinct and temporally dynamic roles in astrocytes and microglia. In the acute phase of stroke, C3a/C3aR signaling exacerbates neuroinflammatory cascades, promoting reactive gliosis and contributing to neuronal injury. Microglia, which expresses the C3aR, responds to ischemic insult by releasing pro-inflammatory cytokines and complement components, including C3, which further amplifies astrocyte activation. This crosstalk drives the transition of astrocytes toward a neurotoxic A1 phenotype via NF-κB-dependent transcriptional programs, as shown by Wang et al. (2024), where microglial-derived IL-1α, TNF, and C1q synergize with C3a/C3aR signaling to induce A1 astrocyte formation and chronic neurodegeneration [30]. Conversely, in the subacute and chronic phases, C3a/C3aR signaling appears to support tissue remodeling and functional recovery. Stokowska et al. (2023) demonstrated that delayed intranasal administration of C3a—initiated seven days post-stroke—attenuates astrocyte reactivity in peri-infarct regions, enhances white matter reorganization, and improves motor outcomes, suggesting a reparative role for C3aR signaling when temporally modulated [20]. Notably, genetic ablation of the C3aR leads to disorganized astrocytic networks and increased microglial density, further underscoring the receptor’s dualistic role depending on the timing and cellular context of activation. These findings highlight the importance of phase-specific targeting of C3a/C3aR signaling in astrocytes and microglia, offering a promising avenue for precision immunomodulation in ischemic stroke therapy. Previous investigations of complement synthesis utilize in vitro models, which may not accurately reflect in vivo conditions, have more recently been augmented by studies across multiple in vivo models and human systems. RNA sequencing analyses confirm that key complement proteins are expressed across principal CNS cell types and are modulated by factors such as injury, infection, developmental signals, and aging [31,32,33]. Additionally, expression of complement components in the CNS exhibits regional and temporal regulation, likely influencing specific component roles and controlling inappropriate downstream effector mechanisms [33,34].

Activated astrocytes predominantly produce C3 [35,36,37], upon stimulation with pro- inflammatory as agents like lipopolysaccharide (LPS). Recent studies have also shown robust upregulation of C3 in microglia across diverse neurodegenerative conditions [35,38], though the impact of this C3 activation remains unclear.

Cell-specific deletion of C3, for example, in microglia-using models like CX3CR1-Cre, enables precise dissection of microglial C3’s role in neurodegeneration, distinct from astrocytic or neuronal sources. Such an approach helps elucidate whether microglial C3 drives synaptic loss, inflammation, or neurotoxicity via pathways like C3a–C3aR and C3b–CR3, offering insight into cell-targeted therapeutic strategies. Expanding this strategy to other CNS and peripheral cell types further delineates the multifaceted roles of C3 in neurodegenerative pathology. Collectively, these compartment-specific approaches offer high-resolution insight into how localized C3 expression contributes to stroke pathology (Table 1).

These genetic models have become indispensable tools for dissecting the multifaceted roles of complement component C3 in ischemic stroke, enabling precise attribution of pathological mechanisms to distinct cellular compartments. The GFAP-Cre astrocyte model has illuminated the contribution of astrocytic C3 to the formation of neurotoxic A1 astrocytes, which exacerbates neuronal injury via NF-κB signaling and complement cascade activation. While this model effectively captures astrogliosis and glial scar dynamics, its limitation lies in the broad expression of GFAP in reactive astrocytes, potentially confounding early developmental versus injury-induced effects. In contrast, the CX3CR1- Cre microglial model offers high specificity for resident microglia and has revealed C3aR- and CR3-mediated synaptic pruning, inflammation, and neurotoxicity following stroke. However, CX3CR1 is also expressed in peripheral monocytes, raising concerns about off-target recombination unless temporally controlled. The CamKIIα- Cre neuronal model has provided insights into autocrine C3 signaling and neuronal vulnerability during excitotoxic stress, particularly through CaMKIIα-mediated pathways. Despite its utility in modeling synaptic remodeling, its restriction to excitatory neurons limits broader neuronal subtype analysis. For vascular contributions, Tie2-Cre and Cdh5-Cre endothelial models have demonstrated that endothelial C3 modulates BBB integrity, leukocyte adhesion, and vascular inflammation. Yet, Tie2-Cre’s pan-endothelial expression can affect hematopoietic lineages, necessitating caution in interpreting CNS-specific effects. The Lyz2-Cre macrophage model has elucidated the role of peripheral macrophage-derived C3 in shaping CNS immune responses and antigen presentation, particularly in border-associated macrophages (BAMs) that influence post-stroke inflammation. However, Lyz2 is expressed in multiple myeloid subsets, which may blur distinctions between BAMs and infiltrating monocytes. Lastly, the CD11c-Cre dendritic cell model has highlighted the role of dendritic cell-derived C3 in CNS immune surveillance and T cell priming, with implications for post-stroke antigen presentation and chronic neuroinflammation. While CD11c is a reliable marker for dendritic cells, its expression in activated microglia and macrophages during inflammation complicates cell-type specificity.

Collectively, these models underscore the cell-type-specific and temporally dynamic roles of C3 in ischemic stroke pathology. Their strategic use enables mechanistic dissection of thrombo-inflammatory injury, neuroimmune crosstalk, and repair processes. However, careful validation of recombination specificity, timing, and off-target effects remains essential for accurate interpretation. These models not only deepen our understanding of stroke pathophysiology but also inform precision-targeted therapeutic strategies aimed at modulating complement signaling in a cell- and phase-specific manner.

## 3. Complement System Dynamics in Innate Immunity and Cerebral Ischemia

The complement system is a key effector of innate immunity and is essential for host defense and tissue homeostasis [44,45]. Composed of over 50 plasma and membrane- bound proteins, the complement system is activated through classical, lectin, and alternative pathways [46]. While historically viewed as primarily responsible for tagging and eliminating microbial intruders, the complement system’s functions are now known to be manifold. Complement is increasingly recognized for roles in immune complex clearance, hematopoietic stem/progenitor cells (HSPCs) mobilization, angiogenesis, synaptic refinement, tissue repair, and lipid regulation [47]. Emerging evidence underscores complement’s multifaceted roles in inflammatory, immune, neurodegenerative, and age-related disorders [6]. Multiple clinical and pre-clinical studies have underscored the role of complement in the pathogenesis and progression of ischemic stroke [5,20,23,48,49,50]. Following cerebral ischemia, complement components are produced not only by infiltrating leukocytes but also locally by activated endothelial cells, neurons, and glial cells, contributing to both injury and repair processes. This dual role of complement establishes the foundation for exploring its temporal dynamics during and after ischemia/reperfusion.

## 4. Role of Complement During and After Ischemia/Reperfusion

During ischemia, ATP is degraded to hypoxanthine, and xanthine dehydrogenase (XDH) is converted to xanthine oxidase (XO), which upon reperfusion reacts with molecular oxygen to produce reactive oxygen species (ROS) such as superoxide and hydrogen peroxide [51]. However, ROS generation following ischemia-reperfusion is multifactorial and not solely dependent on XO activity. Mitochondrial dysfunction, excitotoxicity, and activation of pro-inflammatory transcription factors such as NF-κB also play critical roles by upregulating enzymes like inducible nitric oxide synthase (iNOS), cyclooxygenase-2 (COX-2), and 5-lipoxygenase. These complementary pathways contribute collectively to oxidative damage and neuroinflammation following cerebral ischemia. These ROS activate endothelial cells and stimulate the release of pro-inflammatory cytokines, exacerbating the inflammatory response [52] which may also involve amyloid/tau pathways and glial activation [53]. This process incites secondary injury marked by inflammation oxidative stress, and sustained BBB dysfunction [54,55,56], contributing to further neurodegeneration, functional deficits, and cognitive decline. Cerebral ischemia manifests in an acute phase (within minutes to hours), a subacute phase (within hours to days), and a chronic phase (within days to weeks). Clinical studies report increased serum levels of complement components C4d, C3a, C5a, and C5b-9 during the subacute phase after stroke [57]. Chronic complement activation is reflected by sustained upregulation of the terminal pathway in patient serum seven days post stroke [58,59]. Notably, C5b-9 levels significantly correlate with infarct volume and neurological deficits in stroke patients [57,58].

Stroke is typically modeled in rodents through middle cerebral artery occlusion (MCAO), and in vitro by glucose/oxygen deprivation. These approaches elevate the levels of complement components (C1q, C3a, C5a) in the brain [24,60,61,62], C1q in microglia [63], and C5 in neurons [61]. Immunohistochemical studies suggest both protective and injurious roles of complement activation after stroke [24,60].

C3, the most abundant complement protein in blood, is central to all complement pathways [64,65,66,67]. Upon activation by C3 convertase, it is cleaved into C3a and C3b [68]. C3a promotes inflammation via C3aR, while C3b and its fragments (iC3b, C3dg) engage receptors such as CR1, CR2, CR3, CR4, and CRIg to support pathogen clearance and modulate adaptive immunity [69,70,71,72,73].

Collectively, these findings emphasize the intricate balance by which complement signaling governs both deleterious inflammation and subsequent tissue repair after ischemic insult.

## 5. C3-C3aR Axis in Stroke Pathology and Recovery

Multiple lines of evidence derived from diverse experimental models suggest that complement C3 is central to stroke pathology [23]. While most C3aR-mediated effects after ischemic stroke involve innate CNS cells such as astrocytes, microglia, and neural stem cells, emerging evidence indicates that adaptive immune cells also contribute to post-ischemic pathology. Notably, infiltrating CD3^+^ T cells express C3aRs, suggesting that complement signaling can modulate T cell recruitment, activation, and effector functions within the ischemic brain. This highlights a mechanistic continuum in which C3aR orchestrates both innate and adaptive immune responses, linking early inflammatory processes to subsequent immune-mediated repair or injury. Recent studies have shown that T cells infiltrate the ischemic brain via the blood–brain barrier, choroid plexus, and meningeal routes, and exert both antigen-dependent and antigen-independent effects on neuroinflammation and recovery [74,75]. Although most complement research has focused on innate glial responses, the expression of C3aR on T cells and its role in modulating their phenotype and function—particularly in the post-acute phase—suggests a broader immunological role for complement signaling in stroke pathology [76].

Following MCAO, mRNA levels of the C3a receptor in the ipsilateral brain increase significantly [77], with peaks at 24–48 h post-injury [77,78]. The C3aR is broadly expressed in the CNS, including in astrocytes, microglia, and neural stem cells [79], impacting CNS development [32,80,81] and various neural cell functions. As the C3aR is overexpressed in the subventricular zone and infiltrating CD3+ T cells after MCAO [60], pre- or post-treatment with a low-dose C3aR antagonist (C3aRA, SB290157/JR14a) reduces subcortical infarct volume, decrease mortality, and improves spatial memory and sensory-motor function [82,83,84]. Early C3aRA administration after intracerebral hemorrhage (ICH) accelerates neurological and cognitive recovery in mice, indicating its involvement in both hemorrhagic and ischemic stroke pathogenesis [25]. In vitro, C3aRA treatment of hypoxic endothelial cells reduces cell death, adhesion molecules ICAM1 expression, and p-ERK levels, while increasing occluding, collectively suggesting preserved BBB integrity [85]. Additionally, C3 knockout mice show reduced infarct volume and improved neurological function post MCAO [86], highlighting C3 as a promising therapeutic target in stroke.

However, the widely used C3aRA SB290157 has reported off-target effects and may function as an agonist at high doses [87]. SB290157 has been commonly used as a C3aR antagonist in various disease models including Alzheimer’s Disease [88], intracerebral hemorrhage [25], and stroke [84], to elucidate C3aR-mediated pathways in inflammation and tissue injury. In stroke models, we have shown that its use reduced infarct volume and improved neurological outcomes, suggesting neuroprotective effects [12]. However, its pharmacological reliability is controversial, as studies have shown SB290157 can act as a C3aR agonist in certain systems [89] and exhibit off-target effects, such as altering leukocyte levels [90] independently of the C3aR blockade. This lack of specificity raises concerns about the validity of conclusions drawn solely from its use.

Recent structure–activity relationship studies have identified JR14a, a thiophene-based small molecule, as a potent and selective human C3aR antagonist. JR14a demonstrates an ~100-fold higher potency than SB290157, inhibiting C3a-induced calcium signaling and mast cell degranulation at nanomolar concentrations with minimal cross-reactivity to C5aR. Its favorable metabolic stability and in vivo efficacy highlights its utility as a pharmacological tool for probing C3aR function in inflammatory disease models [91]. Indeed, JR14a markedly attenuated infarct size and improved neurological outcomes when administered after MCAO [84], as well as in photothrombotic and embolic stroke models [83], demonstrating superior efficacy than SB290157. While recent studies have reported that JR14a may also exhibit partial agonist activity under certain in vitro conditions [92,93], its overall robust neuroprotective effects in vivo suggest therapeutic potential warranting further investigation. Beyond its established role in mediating post-ischemic inflammation and immune cell dynamics, emerging evidence suggests that C3aR signaling also contributes to neurogenesis and synaptic plasticity, highlighting its dual function in both the injury and recovery phases of stroke.

## 6. C3aR in Neurogenesis and Plasticity

Beyond its inflammatory role, C3a may enhance post-stroke neurogenesis and plasticity; early evidence shows transient C3aR mRNA downregulation after MCAO [77]. Furthermore, overexpression of C3a under the glial fibrillary acidic protein (GFAP) promoter leads to increased production of GAP43, an axonal marker associated with post-stroke neurite extension [24]. Indeed, C3aR knockout mice showed a reduction in GAP43 indicating that C3a may enhance neurogenesis and synaptic plasticity after stroke [24]. In vitro studies also show that C3a treatment improves astrocyte survival and reduces the expression of GFAP following ischemia [94]. Additionally, intranasal C3a administration one-week post-stroke significantly improved motor function [24].

Reactive gliosis, a coordinated response of astrocytes and microglia, significantly limits tissue damage and restores homeostasis after ischemic injury to the CNS. Specifically, astrocytes play a key role in ischemia-induced plasticity and recovery [95,96,97,98]. Transcriptomic changes in astrocytes vary with context, region, and time [99,100]. Within hours of ischemia onset, astrocytes upregulate GFAP reactivity [4,101]. Days later, astrocytes in the peri-infarct region exhibit cellular hypertrophy, forming a glial scar to limit leukocyte infiltration into healthy tissue [102,103,104]. Microglia are activated within 30 min post-ischemia [105], with their density increasing for weeks [106]. Their expression profile suggests a shift from a neuroprotective to a detrimental phenotype over time [106,107]. Therefore, precise modulation of reactive gliosis holds potential to enhance recovery, but effective pharmacological interventions are currently unavailable. In microglia and astrocytes, C3a is generated through the proteolytic cleavage of C3, upregulates nerve growth factor expression [108,109], enhances astrocyte survival, and reduces GFAP expression following ischemic stress [94]. C3a modulates neural progenitor cell proliferation, migration, and differentiation [24], enhances neurogenesis in naive adult mice [110], and promotes peri-infract cortical neural plasticity after ischemic stroke [24,111]. Conversely, C3aR signaling contributes to Alzheimer’s Disease (AD)-related neurodegeneration [88,112], virus-induced synapse loss and memory impairment [113], and age-associated BBB dysfunction [114].

## 7. Temporal Dynamics of C3aR Activation: Balancing Neurological Injury and Repair

C3aR signaling exerts phase-specific effects in the post-ischemic brain, contributing to both injury and repair. While early complement activation contributes to acute injury, C3a exerts neuroprotective and anti-inflammatory effects during the recovery phase by modulating astrocyte and microglial responses [20,115]. In Alzheimer’s Disease (AD), C3aR activation on astrocytes promotes the release of neurotrophic factors and limits pro-inflammatory signaling, contributing to neuroprotection. In primary microglia, acute C3/C3a exposure enhances phagocytosis, whereas chronic treatment suppresses it; the latter effect is reversed by C3aR antagonism or genetic deletion [116].

C3aR signaling exerts phase-specific effects after stroke: its activation is detrimental in the acute phase but beneficial during recovery [20,76]. Inhibition during the early phase reduces leukocyte-driven inflammation, while delayed C3a administration (from day seven post-stroke) enhances motor recovery, astrocyte function, microglial activity, white matter reorganization, and peri-infarct connectivity—highlighting C3a’s therapeutic potential in stroke rehabilitation. In line with the evolving understanding of complement activation in neurorepair, the phase-specific effects of C3aR activation in ischemic stroke were elucidated using C3aR-deficient (C3aR^−^/^−^) mice and transgenic mice with brain-specific C3a overexpression. In the absence of the C3aR, peri-infarct astrocyte reactivity was significantly elevated, while microglial density was reduced. Conversely, C3a overexpression resulted in diminished astrocyte reactivity and increased microglial presence, suggesting a regulatory role for C3a in glial responses. Importantly, delayed intranasal C3a delivery (day seven post-stroke) enhanced motor recovery, reduced astrocyte reactivity, and spared microglial activation in wild-type mice. In addition, C3a treatment promoted global white matter reorganization and peri-infarct structural connectivity, and upregulated the expression of *Igf1* and *Thbs4*, genes associated with neuronal support and synaptic remodeling. Collectively, these findings indicate that C3aR activation exerts detrimental effects during the acute phase, likely by promoting inflammatory leukocyte recruitment, but supports neurorepair mechanisms during the subacute to chronic phases. This temporal dichotomy underscores the therapeutic potential of targeting C3aR signaling in a phase-specific manner following ischemic stroke [20].

Differential effects of C3aR signaling on functional recovery [20] corroborate a dual role for the C3aR in the post-ischemic brain. During the acute phase, C3aR activation hinders recovery, likely by promoting the recruitment of circulating leukocytes. However, at later stages, the C3aR contributes to motor function improvement by enhancing neural plasticity [24] and white matter reorganization, either through direct effects on neurons or indirectly via reactive gliosis. This suggests that the timing of C3aR-targeted interventions is crucial, with delayed initiation allowing beneficial impacts on astrocytes and neural plasticity while avoiding excessive inflammatory cell and microglial recruitment. C3aR signaling facilitates monocyte recruitment to the prefrontal cortex under chronic stress [117] and promotes leukocyte infiltration into the brain during inflammation [118]. In ischemic stroke, systemic C3aR antagonism reduces granulocyte entry and improves neurological outcomes [60,119]. The opposing effects of constitutive C3aR deficiency and C3a overexpression on early post-stroke recovery highlights its role in leukocyte dynamics.

Despite persistent microgliosis in the peri-infarct cortex for up to eight weeks, delayed intranasal C3a did not alter microglia/macrophage density [20]. Given that monocyte-derived macrophages clear by postnatal day eight (P8) [120], C3a/C3aR-driven changes in Iba1+ cell density at three weeks likely reflect microglial proliferation or migration during the acute phase. This is further supported by divergent Iba1+ responses in C3a-overexpressing mice versus those receiving C3a postnatally, after peak monocyte infiltration on postnatal day seven (P7) [121].

Mechanistically, C3aR activation engages NF-κB [88], PI3K- Akt [122], and ERK1/2 [123] signaling cascades, leading to transcriptional changes that support tissue repair. In astrocytes, C3aR stimulation upregulates IGF-1 and Thbs4, enhancing neurotrophic support and glial scar modulation to preserve neuronal networks after ischemia [20]. Astrocyte-driven C3 release in Alzheimer’s Disease activates microglial and neuronal C3aRs to regulate phagocytosis and synaptic integrity, with acute C3aR engagement enhancing clearance but chronic signaling impairing it [116]. This parallels post-ischemic C3aR’s dual roles in stroke and underscores the need for phase-specific modulation, including timed C3aR antagonism under sustained inflammation. C3aR antagonism may restore brain-derived neurotrophic factor (BDNF)-dependent neurogenesis, synaptic homeostasis, and Notch/Wnt signaling via the ATP–adenosine–NO pathway [124].

Accumulating evidence suggests that the effects of C3a-C3aR signaling in the post-ischemic brain are not only temporally regulated but also shaped by spatial context and the *intensity* of receptor activation. Although systematic quantitative mapping of C3aR signaling intensity across different brain regions after ischemic stroke is currently lacking, existing studies allow us to infer region- and phase-specific roles. The infarct core—exposed to severe ischemia and irreversible damage—likely exhibits intense and uncontrolled complement activation, which may exacerbate neuronal injury through recruitment of inflammatory leukocytes, glial activation, and breakdown of the blood–brain barrier. In contrast, the peri-infarct (penumbral) zones may experience more moderate levels of C3aR activation, which could support protective responses such as phagocytosis of cellular debris, modulation of glial phenotypes, and promotion of repair pathways.

This dualistic nature of C3aR signaling suggests the neurotoxic potential of complement activation in the acute phase of stroke, and the reparative influence of controlled activation during the subacute and chronic phases [76]. Further, the administration of C3a in the subacute phase post-stroke promoting functional recovery by stimulating astrocyte-derived IGF-1, suggests a dose- and context-dependent protective role for C3aR signaling during recovery [20].

Together, these observations suggest that the degree of C3aR stimulation may serve as a switch between deleterious and beneficial outcomes. Excessive, early phase activation—particularly in the infarct core—may drive neuroinflammation and tissue damage, whereas moderate, spatially restricted activation during later phases may facilitate glial-vascular remodeling and neurorepair. These insights support a model of complement action in stroke that is not only temporally and anatomically regulated but also dependent on the intensity of signaling (Table 2). Future studies employing spatial transcriptomics and quantitative receptor signaling assays are needed to elucidate these gradients of C3aR activity and refine therapeutic approaches that exploit this duality. Building on the temporally and spatially regulated roles of C3aR signaling in adult stroke recovery, emerging evidence suggests that similar mechanisms may operate in the immature brain, where complement-mediated modulation of neuroinflammation and gliosis could critically influence outcomes in neonatal hypoxic-ischemic encephalopathy (HIE).

## 8. Extending C3a/C3aR-Mediated Neuroprotection to Neonatal Hypoxic-Ischemic Encephalopathy (HIE)

While majority of studies on complement activation in neuroinflammation have focused on adult ischemic stroke, the mechanisms governing complement-mediated injury and repair may be conserved, or even amplified, in the developing brain. Neonatal HIE shares key pathophysiological features with stroke, including excitotoxicity, oxidative stress, and inflammation, but involves distinct age-dependent responses and cellular susceptibility [128,129,130]. Exploring C3a/C3aR signaling in this context reveals critical insights into its role in neurodevelopment and injury resolution.

Astrocyte-targeted C3a expression mitigates hippocampal neurodegeneration and reactive gliosis after HIE [21]. Conversely, genetic deletion of the C3aR exacerbates neuronal loss and is associated with increased microglial density, suggesting that astrocyte-derived C3a exerts a protective effect in the neonatal brain by modulating neuroinflammatory responses. These findings reinforce the concept that C3a/C3aR signaling can promote neuroprotection and repair when precisely modulated in a disease stage and cell type-specific context [21]. Intranasal delivery of C3a following injury markedly attenuated hippocampal neurodegeneration and reactive gliosis in wild-type mice [21]. Collectively, these results suggest that neonatal hypoxic-ischemic brain injury triggers persistent neurodegeneration, which can be significantly mitigated by C3aR agonists—potentially via regulation of reactive gliosis. C3a/C3aR signaling contributes to neuroprotection in neonatal HIE by promoting neuronal maturation, neurite outgrowth, and NGF expression [21,131]. While therapeutic hypothermia increases C3aR expression without affecting early glial or neuronal density, intranasal C3a administration reduces neurodegeneration and improves cognitive outcomes, suggesting its potential as an adjunct therapy. Therapeutic hypothermia—the sole clinically validated intervention for improving outcomes in neonates with HIE [132]—coincidentally upregulates C3aR expression in the rat brain at 24 and 48 h following hypoxia-ischemia (HI) [131]. Notably, this treatment does not alter neuronal, microglial, or astrocytic density during the early post-HI period [131]. C3a activation enhances nerve growth factor expression in microglia [133], a broadly neuroprotective factor in neonatal HI models [134] that also promotes axonal growth and branching both in vitro and in the adult brain [135,136]. Additionally, C3a directly facilitates neurite outgrowth and neuronal maturation [137]. Importantly, intranasal C3a treatment reduces neurodegeneration and mitigated HI-induced cognitive deficits [138] highlighting its potential as an adjunct therapy to hypothermia.

While the neuroprotective effects of C3a/C3aR signaling in neonatal hypoxic-ischemic injury underscores its developmental and cell-specific relevance, a deeper understanding of its interplay with the coagulation cascade reveals how complement activation interfaces with vascular integrity and thrombo-inflammatory injury across the lifespan.

## 9. Interplay Between Complement and Coagulation Systems

The complement and coagulation systems are evolutionarily connected and functionally intertwined, maintaining homeostasis through functionally distinct but synergistic roles: the complement system mediates immune surveillance and clearance of cellular debris, while the coagulation cascade ensures vascular integrity and tissue repair following injury [139]. Notably, complement serves as a critical interface between innate immunity and coagulation [140]. A bidirectional crosstalk between these systems, particularly under pathological conditions, contributes to complications such as thrombosis in various diseases including stroke. Thrombosis plays a significant role in post-ischemic brain injury, as demonstrated in multiple studies focusing on the role of mannose-binding lectin (MBL). de la Rosa et al. (2014) showed that MBL promotes local microvascular thrombosis following transient cerebral ischemia, suggesting its early contribution to ischemic damage [141]. In another study, Orsini et al. (2012) reported that targeting MBL provided sustained neuroprotection, even when treatment was delayed, highlighting MBL’s critical role in thrombosis and downstream injury [142]. More recently, Orsini et al. (2018) further elucidated that MBL drives a platelet pro-inflammatory phenotype and vascular damage post-stroke through IL-1α signaling, reinforcing the link between MBL-mediated thrombosis and inflammation in the ischemic brain [143]. Together, these studies underscore thrombosis, particularly MBL-mediated, as a key contributor to post-ischemic brain injury.

The complement system triggers both inflammation and thrombosis. Specifically, C3a binding to the C3aR mediates various pro-inflammatory and prothrombotic responses. Complement promotes thrombogenesis by enhancing inflammation and coagulation efficiency [144], activating platelets, upregulating tissue factor (TF) expression, modulating mast cell and basophil responses, and triggering TF-mediated coagulation via membrane attack complex (MAC)-induced phospholipid remodeling [145,146,147,148].

Dysregulated complement activation can result in irreversible tissue or organ damage; thus, multiple regulatory proteins tightly control the system to prevent excessive inflammation and injury [149]. Similarly, the coagulation cascade is a homeostatic system triggered by vascular injury, comprising cellular and proteolytic components organized into intrinsic, extrinsic, and common pathways [150]. Its crosstalk with complement involves bidirectional regulation [151]. Anaphylatoxins C3a and C5a activate and aggregate platelets [152], which in turn initiate classical and alternative complement pathways [153]. Platelet-derived Factor H, released upon thrombin stimulation or C3b binding [154], co-purifies with thrombospondin-1 [155], serves as a substrate for FXIIIa [156], and may localize to clot sites. Factor H inhibits FXI activation by thrombin or FXIIa [157], but is cleaved by FXIa, impairing its regulatory function [158]. C1 inhibitor suppresses FXIIa [159], while MASP-2 cleaves prothrombin [160], and MAC (C5b-9) promotes thrombin generation independently of Factor V [161]. C5a enhances tissue factor expression in endothelial cells and neutrophils [162] and induces PAI-1–mediated prothrombotic responses in mast cells and basophils [145]. Thrombin and plasmin cleave C3 and C5 independently of canonical pathways [163,164], and FIXa, FXa, FXIa, and plasmin generate C3a and C5a in vitro [165]. FXIIa also activates the classical pathway via the C1 complex [166].

During ischemic stroke, coagulation and innate brain immunity concomitantly impact the extent of injury and repair. Vascular occlusion and endothelial damage trigger thrombin generation and fibrin deposition, which not only exacerbate microvascular obstruction but also activate protease-activated receptors (PARs) on neurons and glia, driving inflammation and blood–brain barrier (BBB) breakdown [167,168]. Simultaneously, resident CNS cells—including astrocytes, microglia, neurons and endothelium—upregulate the synthesis of complement proteins in response to danger signals. Locally produced C3 is cleaved into C3a, which binds to the C3aR on microglia and astrocytes to orchestrate inflammatory cytokine release, phagocytosis, and later repair processes. Whereas systemic complement influx through a leaky BBB can amplify global inflammatory cascades, intracerebral complement synthesis allows precise, regionally tailored modulation of tissue responses promoting debris clearance and neurovascular remodeling in the penumbra. Thus, coagulation factors primarily perpetuate acute vascular and thrombo-inflammatory injury, whereas brain-intrinsic complement production likely provides both injurious and reparative signals in a spatiotemporally controlled fashion.

Given the intricate crosstalk between complement and coagulation systems in driving thrombo-inflammatory injury during stroke, understanding how fibrinolytic agents such as tPA and TNK influence complement activation—particularly C3a generation—offers critical insight into the unintended neurovascular consequences of reperfusion therapy and the potential for targeted complement modulation to mitigate these effects.

## 10. Targeting Complement Pathways During Fibrinolysis to Mitigate Brain Injury in Stroke

Intravenous tissue plasminogen activator (tPA) and more recently Tenecteplase (TNK), which catalyze plasminogen-to-plasmin conversion to promote fibrinolysis, remain the only approved pharmacologic treatments for acute ischemic stroke. Although timely thrombolysis can limit infarct progression and improve outcomes, its broader use is constrained by the risk of intracerebral hemorrhage. The neurotoxic effects of tPA are well documented, with early studies demonstrating its pro-apoptotic impact on cultured neurons, ischemic brain endothelium, and mouse cortical neurons [169]. tPA also worsens post-ischemic neuronal injury in both wild-type and tPA-deficient mice [170] and its absence mitigates cortical damage and edema following traumatic brain injury [171].

In addition to their established neurotoxic effects [170,172], tPA and Tenecteplase may exacerbate injury by promoting complement activation through plasmin-mediated cleavage. Complement activation extends beyond the classical, lectin, and alternative pathways to include extrinsic mechanisms as well.

Notably, thrombin can directly cleave C5 independent of C3 [163], and plasmin cleaves both C3 and C5 in vitro [173]. However, the relevance of these pathways in vivo remains incompletely understood. Plasma complement activation is increased post-tPA during coronary thrombolysis [174]. Our prior findings showed that tPA markedly promotes C3 cleavage in the ischemic brain [175]. Notably, tPA induces substantial C3a generation even in mannose-binding lectin (MBL)-deficient mice, which lack ischemia-driven complement activation [176], indicating an MBL-independent mechanism. Notably, MBL facilitates microvascular thrombus formation after transient cerebral ischemia and worsens reperfusion injury [141]. In contrast, elevated C3a levels following tPA in WT mice suggest that C3aRA may confer protection in this context. Remarkably, tPA also induces C3 cleavage in non-ischemic WT brains, suggesting a possible role in disrupting blood–brain barrier integrity [175]. In vitro, tPA-driven C3 cleavage proceeds through plasmin, as α2-antiplasmin abrogates C3a production. Lack of suppression by CD35 further supports that this process bypasses the canonical C3 convertase. The upregulation of plasminogen receptors at injury sites [177] supports the hypothesis that tPA facilitates local plasmin generation and C3 cleavage at the ischemic endothelium. Our findings further confirmed the neuroprotective efficacy of complement inhibition in stroke and represent the first to evaluate its functional impact in conjunction with tPA therapy [60,86,119,176,178,179].

Despite reduced infarct volume in tPA-treated MCAO [180] and the thromboembolic model [181] of stroke, likely due to improved reperfusion, C3a levels rise significantly in ischemic brain tissue, accompanied by exacerbated brain edema and hemorrhage, mirroring clinical outcomes in stroke patients [182]. While the role of C3a in stroke-induced edema remains uncharacterized, other models showed its involvement in inflammatory cell infiltration and vascular permeability [183]. The hemorrhagic transformation may result from reperfusion through permeable micro vessels, allowing for red blood cell extravasation [184]. Further studies are needed to elucidate to what extent the treatment with C3aRA mitigates edema and hemorrhage. While experimental evidence supports the neuroprotective potential of complement inhibition—particularly C3aR antagonism—in the context of thrombolytic therapy, translating these findings into clinical practice demands a nuanced understanding of C3aR’s phase-specific roles, pharmacological limitations, and relevance across diverse patient populations.

## 11. Translational Potential and Limitations of Targeting the C3aR in Ischemic Stroke

Preclinical studies demonstrate that antagonism or genetic deletion of the C3aR confers neuroprotection in experimental stroke models—by reducing infarct volume, edema, neuroinflammation, and preserving BBB integrity—but translational challenges remain significant [20]. Notably, C3aR signaling displays dual and context-dependent roles; it can exacerbate neuroinflammation and promote injury in acute phase, it may also support repair during the subacute and chronic phases through astrocyte modulation, neuronal plasticity, and white matter organization [20,76]. The pleiotropic nature of C3aR signaling complicates therapeutic timing and dosage optimization [185]. Furthermore, most studies use young male rodents, limiting relevance to aged, comorbid human populations typically affected by stroke [186,187]. No C3aR-targeting agents have yet entered clinical trials for stroke, and concerns persist regarding off-target effects of currently available antagonists such as SB290157, which shows partial agonist effects in immune and glial cells [188]. Newer compounds (e.g., JR14a) show improved efficacy in animal models, but their pharmacokinetics, dosing, and safety in humans remain untested [91]. Thus, although promising, translation of C3aR-based therapies requires careful evaluation of therapeutic windows, cell-specific roles, and broader safety profiles. Given the translational hurdles posed by C3aR’s pleiotropic signaling and pharmacological constraints, future research must prioritize mechanistic precision and therapeutic refinement through advanced models and molecular tools to unlock the full potential of complement-targeted interventions in stroke recovery.

## 12. Future Directions for Studies of Complement Activation in Stroke

Future studies should leverage in vivo models, including cell-specific complement knockout mice, to delineate the temporal and spatial dynamics of complement activation in stroke. Investigating the role of individual complement pathways across distinct phases of ischemic injury will inform timing and specificity of therapeutic interventions. Focus should be placed on identifying complement-mediated mechanisms that confer neuroprotection while limiting maladaptive inflammation. Combining complement inhibitors with established neuroprotective agents may yield synergistic benefits. Advanced molecular imaging and transcriptomic tools can elucidate complement activity in discrete brain regions and cell types. Finally, long-term studies in chronic stroke models are needed to evaluate the sustained impact of complement modulation on neurological and cognitive recovery.

## 13. Conclusions

The complement system plays a dual role in ischemic stroke, contributing to both injury and repair processes. While historically considered a mediator of neuroinflammation and tissue damage, growing evidence suggests that complement components, particularly C3 and its receptor, C3aR, also participate in neuroprotection and post-stroke regeneration. The effects of complement activation appear to be highly context-dependent- shaped by timing, anatomical localization, and activation intensity. Targeting C3aR signaling has shown promise in mitigating acute ischemic injury and supporting recovery in preclinical models. However, translating these findings into clinical practice remains complex, particularly given the multifactorial nature of stroke. A notable limitation in the current literature is the predominant use of non-aged male mice, which does not reflect the sex- and age-related variability observed in human stroke and complement responses. Moreover, while small-molecule C3aR antagonists such as SB290157 and JR14a have demonstrated neuroprotective effects in experimental models, their pharmacological profiles include potential off-target activities that may confound interpretation. These considerations reinforce the importance of using conditional C3aR knockout models—to better delineate the receptor’s specific role in stroke pathophysiology. Future investigations should aim to include both sexes and aged-animal models to enhance the translational relevance of complement-targeted therapies in stroke.

## Figures and Tables

**Table 1:** Cell-specific C3 knockout strategies and relevance to stroke pathology.

Cell Type/Tissue	Cre Line (KO Model)	Hypothesized Role of C3	Relevant Stroke Pathology
Astrocytes [39]	GFAP-Cre	Drives formation of neurotoxic A1 astrocytes; contributes to neuronal damage	Astrogliosis, neurotoxicity
Microglia [40]	CX3CR1-Cre	Mediates synaptic loss, inflammation, and neurotoxicity (via C3aR and CR3 pathways)	Microgliosis, inflammation
Neurons [41]	CamKIIα-Cre	Modulates neuronal vulnerability and autocrine signaling during injury	Neurodegeneration, synaptic remodeling
Endothelial Cells [42]	Tie2-Cre/Cdh5-Cre	Regulates BBB integrity, leukocyte adhesion, and vascular inflammation	BBB breakdown, vascular inflammation
Peripheral Macrophages [43]	Lyz2-Cre	Shapes CNS immune responses and contributes to antigen presentation	Peripheral immune infiltration
Dendritic Cells [43]	CD11c-Cre	Modulates CNS immune surveillance and inflammation	Immune modulation and antigen presentation

**Table 2:** Temporal dynamics of C3aR activation in CNS injury.

Phase	Timing	C3aR Activation Effects	Key Mechanisms and Outcomes	References
Acute (injurious)	Minutes to hours post-ischemia	Exacerbates BBB disruption, promotes endothelial inflammation and microvascular thrombosis	Endothelial C3aR triggers VCAM-1 upregulation, Ca^2+^ influx, junction disruption and leukocyte infiltration, driving edema and injury	[20,21,24,76,84,114,125,126]
Subacute/early reparative	Hours to days post-ischemia	Supports clearance of cellular debris, reduces glial inflammation, limits necrosis	Microglial/astrocyte C3 signaling via C3aR enhances efferocytosis and curtails reactive gliosis in developing cerebellum	[125,126,127]
Chronic (reparative)	Days to weeks post-ischemia	Promotes neurogenesis, synaptic remodeling, and functional recovery	C3aR signaling stimulates neurogenesis in adult brain post-stroke, neural progenitor proliferation and plasticity	[24,76]

## Data Availability

No new data were created or analyzed in this study.
